# ELISA-Based Analysis Reveals an Anti-SARS-CoV-2 Protein Immune Response Profile Associated with Disease Severity

**DOI:** 10.3390/jcm11020405

**Published:** 2022-01-14

**Authors:** Charline Herrscher, Sébastien Eymieux, Christophe Gaborit, Hélène Blasco, Julien Marlet, Karl Stefic, Philippe Roingeard, Leslie Grammatico-Guillon, Christophe Hourioux

**Affiliations:** 1 Inserm U1259 Morphogénèse et Antigénicité du VIH et des virus des Hépatites, Université de Tours et CHRU de Tours, 37000 Tours, France; julien.marlet@univ-tours.fr (J.M.); karl.stefic@univ-tours.fr (K.S.); philippe.roingeard@univ-tours.fr (P.R.); leslie.guillon@univ-tours.fr (L.G.-G.); christophe.hourioux@univ-tours.fr (C.H.); 2 Epidémiologie des Données Cliniques en Centre-Val de Loire (EpiDcliC), Centre Hospitalier Universitaire de Tours, 37000 Tours, France; christophe.gaborit@univ-tours.fr; 3 Service de Biochimie et Biologie Moléculaire, CHRU de Tours, 37000 Tours, France; helene.blasco@univ-tours.fr; 4 Service de Bactériologie-Virologie-Hygiène, CHRU de Tours, 37000 Tours, France

**Keywords:** SARS-CoV-2 antibodies, SARS-CoV-2 linear epitopes, COVID-19, disease severity

## Abstract

Since the start of the COVID-19 pandemic, many studies have investigated the humoral response to SARS-CoV-2 during infection. Studies with native viral proteins constitute a first-line approach to assessing the overall immune response, but small peptides are an accurate and valuable tool for the fine characterization of B-cell epitopes, despite the restriction of this approach to the determination of linear epitopes. In this study, we used ELISA and peptides covering a selection of structural and non-structural SARS-CoV-2 proteins to identify key epitopes eliciting a strong immune response that could serve as a biological signature of disease characteristics, such as severity, in particular. We used 213 plasma samples from a cohort of patients well-characterized clinically and biologically and followed for COVID-19 infection. We found that patients developing severe disease had higher titers of antibodies mapping to multiple specific epitopes than patients with mild to moderate disease. These data are potentially important as they could be used for immunological profiling to improve our knowledge of the quantitative and qualitative characteristics of the humoral response in relation to patient outcome.

## 1. Introduction

In December 2019, cases of atypical pneumonia were reported in Wuhan, China. The unknown etiologic agent was later identified as a new coronavirus displaying 79.6% of genomic sequence identity to the severe acute respiratory syndrome coronavirus (SARS-CoV) [1]. In March 2020, the World Health Organization (WHO) declared that coronavirus induced disease 2019 (COVID-19) was a global pandemic. During the first 24 months of the pandemic, there have been more than 280 million laboratory-confirmed cases of COVID-19 with over five million deaths.

Severe acute respiratory syndrome coronavirus-2 (SARS-CoV-2) is an enveloped, single-stranded positive sense RNA virus from the genus *Betacoronavirus* [2]. The main manifestations of SARS-CoV-2 infection include respiratory symptoms, systemic inflammation leading to multi-organ dysfunction, such as acute respiratory distress syndrome, cardiovascular disorders and neurological symptoms [3,4,5,6]. SARS-CoV-2 is more contagious than SARS-CoV, its greater transmissibility possibly being due to the larger number of asymptomatic patients with a high viral load [7]. The viral genome encodes four main structural proteins—spike (S), envelope (E), membrane (M) and nucleocapsid (N)—essential for virion assembly and infection. Spike is the outermost protein on the surface of the virus. It contains a receptor-binding domain (RBD) that interacts with the host receptor, angiotensin-converting enzyme 2 (ACE2), to mediate viral entry into cells [8]. The M protein is the most abundant structural protein of SARS-CoV-2 and is able to bind all the other structural proteins. Its function remains incompletely understood, but the binding of M protein has been shown to stabilize the N protein and to foster viral assembly by stabilizing the N protein-RNA complex [9]. The E protein is the smallest of the structural proteins playing an important role in virus assembly, release and virulence [10]. The N protein is highly conserved, with an amino-acid sequence 90% identical to that of the SARS-CoV nucleocapsid [11]. The N protein packages the RNA of the viral genome and participates in virion assembly through its interaction with the M protein [12]. The N proteins of many coronaviruses are highly immunogenic and produced in abundance in virus-infected cells [13].

Much attention has been devoted to identifying the immunodominant linear epitopes on SARS-CoV-2 proteins since the start of the outbreak. These epitopes are important for diagnosis, for the development of monoclonal antibodies for prevention and treatment, and for the design of peptide-based vaccines [14,15,16]. Several immunodominant linear epitopes have been identified on the S, M, E, N (for review [17]) and ORF8 [18] proteins. However, only a few studies have investigated the possible correlation between specific reactivity to particular linear epitopes and disease severity [19,20,21]. Such correlations may provide important information about the pathogenesis of SARS-CoV-2 infection and are of potential utility for patient stratification in medical practice. In this study, we performed bioinformatics analysis to predict antigenic linear epitopes in the S, M, N and ORF8 proteins, which we then used to establish peptide-based ELISAs for use on plasma samples from COVID-19 patients and controls. We then investigated the correlations between severity and reactivity to several of the epitopes identified.

## 2. Materials and Methods

### 2.1. Design and Participants

We performed a cross-sectional study of patients testing positive for SARS-CoV-2 by RT-PCR during their hospitalization at Tours Regional University Hospital (Loire Valley, France) between 1 April 2020 and 1 July 2021. We analyzed plasma samples collected from these patients 25–35 days after symptom onset. We excluded: (i) patients who refused to participate (ii) patients for whom no clinical data were available and (iii) patients with incomplete biological data.

### 2.2. Clinical Variables of Interest

The outcomes for the study population were analyzed according to patient characteristics, including sociodemographic factors (age, sex) and comorbid conditions (cardiovascular disease, hypertension, diabetes mellitus, lung disease, renal insufficiency, dialysis, kidney transplantation, liver failure and obesity). Participants were classified into three groups (mild, moderate and severe) according to the WHO COVID-19 classification of cases [22].

### 2.3. Linear B-Cell Epitope Prediction

We used the primary sequences of the S, N, M and ORF8 proteins from the original (Wuhan) SARS-CoV-2 strain and the Bepipred-2.0 prediction module (https://services.healthtech.dtu.dk/service.php?BepiPred-2.0, accessed on 13 December 2021) to identify likely B-cell linear epitopes in these proteins [23]. The epitope threshold value was set at 0.55. We also used the Ellipro Antibody epitope prediction tool (http://tools.iedb.org/ellipro/, accessed on 13 December 2021) [24] with the 3D structure prediction files for the S protein (PDB ID: 6VXX and 6VSB), the N-terminal domain of the N protein (PDB ID: 7CDZ) and the ORF 8 protein (PDB ID: 7JX6) to identify complementary B-linear epitopes, and for the formal exclusion of conformational epitopes from our peptide set. We also excluded predicted sequences covering a predicted N-glycosylation site in the S protein, because chemical synthesis does not allow the addition of these glycosylated motifs to the peptide sequence. Long sequences predicted to be linear epitopes were split into peptides overlapping by five amino acids. Finally, based on the high degree of sequence identity between the SARS-CoV and SARS-CoV-2 N proteins, we were able to confirm our N peptide set on the basis of experimental determination of peptide reactivity in a SARS-CoV peptide-based ELISA described in a previous study [25]. N proteins display significant conservation among human coronaviruses. We therefore limited cross-reactivity with antibodies against commonly circulating human coronaviruses by aligning the sequences of the SARS-CoV-2 nucleocapsid and its homologs in HCoV-HUK1, HCoV-OC43, HCoV-NL63 and HCoV-229E, to identify immunogenic peptides with the lowest degree of similarity.

### 2.4. Linear Peptide Library

Based on Bepipred predictions, we had 60 linear peptides (19–38 residues) synthesized (Proteogenix, Schiltigheim, France) (Table S1). Forty-six peptides (SK1 to SK39) based on the primary sequence of the S protein were synthesized. Ten linear peptides (NC1 to NC8) were synthesized for the N protein, and one peptide was synthesized for the M protein. Three peptides (NS1 to NS3) were synthesized based on the sequence of the ORF8 protein. This set of peptides included some peptides covering two versions of the same region. Peptide names ending in an apostrophe correspond to an extended version of the peptide to which 10 additional amino acids were added relative to the native peptide, equally distributed between the N-terminal and C-terminal parts of the molecule. A scrambled peptide (SK36) was used as the blank. The synthetic peptides were then purified by reverse-phase HPLC (>80% purity), with verification of their molecular weight by mass spectrometry. The purified peptides were then dissolved to generate a 1 mg/mL solution, in accordance with the manufacturer’s instructions, for storage at temperature below −20 °C.

### 2.5. Peptide-Base ELISA

Synthesized peptides were prepared in sterile phosphate-buffered saline (PBS, Thermo Fisher Scientific, Waltham, MA, USA) at a final concentration of 3000 ng/mL. Nunc Maxisorp 96-well immunoassay plates (Thermo Fisher Scientific) were coated by overnight incubation at 4 °C with 100 μL of prepared peptides per well. The plates were washed three times with a plate washer. The washing solution (PBST) used for all washes was composed PBS supplemented with 0.1% Tween-20 (Sigma Aldrich, St. Louis, MO, USA). The plates were then blocked by incubation with 3% non-fat dry milk (NFDM) prepared in PBS at room temperature for 1 h. The plates were washed once and 100 µL of diluted (1:50 in 1% NFDM prepared in PBST) human plasma were added per well. Then plates were incubated at room temperature for 30 min, washed six times with washing solution and incubated at room temperature for 30 min with 100 µL horseradish peroxidase (HRP)-labeled mouse anti-human IgG Fcy-specific antibody (Southern Biotech, Birmingham, AL, USA) diluted 1:10,000 in 1% NFDM prepared in PBST. The plates were washed six times and incubated with 100 µL developing solution per well (Sigma Fast OPD, Sigma Aldrich) for 10 min in the dark at room temperature. The reaction was stopped by adding 100 µL 2 N H_2_SO_4_ per well and absorbance was measured on a microplate reader at 490 nm, with 630 nm as the reference wavelength.

### 2.6. Peptides of Interest

We pooled 50 different pre-pandemic plasma samples collected before January 2018, to constitute a pooled negative control (unrelated to the COVID-19 patients further investigated in our study). We first used 50 plasma samples randomly selected from the COVID-19 patients described above for the selection of peptides of interest. Briefly, all plasma samples (negative controls and samples from COVID-19 patients) were tested in duplicate against the whole set of synthesized peptides using ELISA assay.

### 2.7. Statistical Analysis

The continuous variables are expressed as the mean ± standard deviation (SD), whereas the qualitative variables are expressed as absolute numbers and percentages. Peptide OD values were initially studied as continuous variables, but sensitivity analyses were performed to assess the value of treating peptide OD as a qualitative variable with a relevant threshold identified from ROC curves and graphical presentations. An optimal cut-off value was identified for each peptide. Each cut-off has been chosen accordingly to the best balance between odds ratio of severity and its *p*-value. We proposed a graphical plot that illustrates the OD ability to classify the COVID-19 disease as severe according to the variations in the OD value distribution, giving a discrimination threshold = cut-off (on the same basis as ROC curves) (Supplementary Data Figure S1). For the identification of factors associated with severity, we first performed bivariate analyses with the variables of interest, including the OD for each peptide, clinical and sociodemographic variables, in univariate logistic regression models. We then performed multiple logistic regression, including variables for which *p* < 0.2 in the model, together with variables considered clinically relevant. A descending stepwise process was used to select the final linear regression model. The odd ratios (ORs) and 95% CIs were estimated for an association between COVID-19 severity and biological outcomes, including the OD of each peptide as a binary variable with a threshold/cut-off chosen on the basis of OD distribution and OR.

All tests were two-tailed, and *p* values below 0.05 were considered statistically significant. We included all participants for whom the variables of interest were available in the final analysis, without imputing missing data. All statistical analysis were performed with SAS, version 9.4, with SAS Enterprise Guide 71 64-bit (SAS Institute Inc., Cary, NC, USA).

### 2.8. Ethics Approval

This study involved the reuse of data that had already been recorded. It falls within the scope of the French Research Commission according to the 2016—41 law of 26 January 2016 on the modernization of the French health system (DC 2020_097), according to which written informed consent is required from all individuals included in biomedical studies. Samples were obtained from the registered biological collection DC-2020-3961.

## 3. Results

### 3.1. Characteristics of Participants

During the study period, we obtained 213 plasma samples from SARS-CoV-2 positive patients for whom complete clinical and biological data were available, and who had given consent for participation. The sociodemographic and clinical characteristics of the participants are shown in Table 1.

The median age of the participants was 69 (55–84) years, with a sex ratio of 0.85 (99 (46%) men and 114 (54%) women). The most common comorbid condition was hypertension (109 patients, 51%), followed by cardiovascular diseases (76 patients, 35%), obesity (52 patients, 24%) and diabetes mellitus (44 patients, 21%). Eighty-four patients (39%) had mild infections, 42 patients (20%) developed moderate infections and 87 participants (41%) were admitted to the intensive care unit (ICU) due to severe infection. Among patients developing mild infections, the main comorbid condition was hypertension (34%), followed by cardiovascular diseases (25%) and diabetes mellitus (20%). The proportion of women was higher than the proportion of men in the group of patients with mild infection (68% versus 32% in men). The most frequent comorbid factors in patients with moderate infection were hypertension (60%), cardiovascular diseases (43%) and obesity (24%). The other comorbid conditions were evenly distributed between disease severity groups (around 20% in each group). The proportion of women was higher than the proportion of men in the group of patients with moderate infections (64% versus 36% in men). Among patients admitted to the ICU, the percentage of patients with comorbidity factors was high, whatever the comorbid conditions considered. Hypertension (63%), cardiovascular disease (42%) and obesity were the most common comorbid conditions. The proportion of men was higher than the proportion of women in the severe infection group (66% versus 34% in women).

### 3.2. Selecting the Peptides of Interest

**Figure 1:**
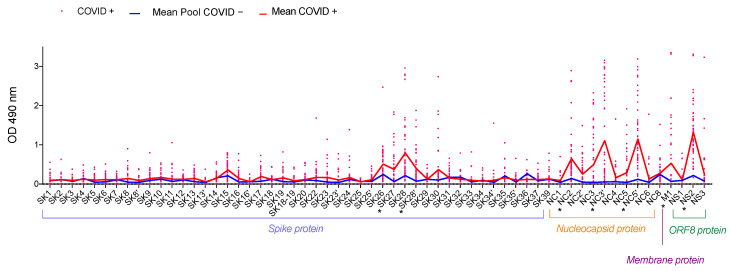
Identification of IgG-specific B-cell epitopes on the spike (S), nucleocapsid (N), membrane (M) and ORF8 proteins. We pooled 50 pre-pandemic plasma samples collected before January 2018 to constitute a COVID-19 negative control (Pool COVID−). We used 50 plasma samples from patients tested positive for SARS-CoV-2 by RT-PCR, collected between 25 and 35 days after the onset of symptom, to evaluate IgG reactivity to 60 peptides. OD results are represented for each sample as a pink point for COVID+ samples. The red line represents the mean OD for COVID+ patients and the blue line represents the mean OD of 50 repeated measures for the COVID− pool. * indicates the peptides selected for further analysis.

Our peptide mapping identified several hotspots in the SARS-CoV-2 proteins covered (Figure 1). In this first set of ELISAs, peptides giving a mean optical density (OD) greater than 0.3 and at least five times higher than the OD obtained with the negative control were selected for further analysis (Table S2).

For the S protein, at least one region was found to constitute a B-cell epitope. This region was covered by peptides SK27 and SK28. The mean OD obtained with SK27 was 0.335 for COVID+ versus 0.024 for the COVID− pool. For SK28, the mean OD for COVID+ samples was 0.748 and that for the COVID− pool was 0.175. Peptide reactivity was greater for the N protein than for the S protein. Three peptides reacted strongly with COVID+ samples: NC2, NC3′ and NC5′. Mean OD was 0.605 for NC2 vs. 0.101 for the COVID− pool, 1.063 for NC3′ vs. 0.017 for the COVID− pool and 1.103 for NC5′ vs. 0.082 for the COVID− pool. The single peptide obtained from the M protein showed also displayed a large difference in reactivity between the COVID+ and COVID− pool samples (0.489 vs. 0.032, respectively). For ORF8, only one peptide was highly reactive with COVID+ samples: NS2, with a mean OD of 1.275, versus 0.176 for the COVID− pool. We therefore selected the following peptides (SK27, SK28, NC2, NC3′, NC5′, M1 and NS2) for the next step of the study.

### 3.3. Analysis of the Selected Peptides

**Figure 2:**
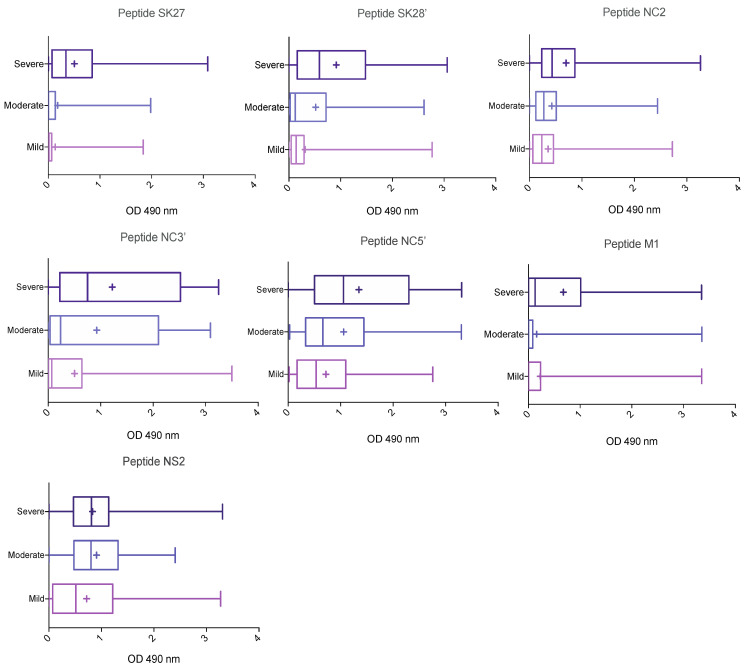
Association of epitope-specific IgG levels with disease severity. COVID-19 patients were classified on the basis of the clinical severity of disease (mild: *n* = 84; moderate: *n* = 42 and severe/ICU: *n* = 87).

We analyzed antibody responses to selected peptides according to disease severity. The median OD and interquartile range for each peptide are shown in Table 2, and a boxplot for each peptide against disease severity is shown in Figure 2.

These results suggest that the IgG response to selected epitopes, other than NS2, was stronger in patients with severe COVID-19 than in those with mild or moderate disease. Indeed, for every peptide other than NS2, the median value increased with disease severity. Large differences between groups were observed for some epitopes. Reactivity to the M1 peptide differed considerably between the mild, moderate and severe COVID-19 groups (Figure 2). By contrast, there was little difference between the groups in terms of reactivity to the NC2′ peptide. For confirmation of these observations, we used logistic modeling to analyze the data, to identify clinical variables and peptides associated with disease severity.

### 3.4. Analysis of Variables and Peptides Associated with Disease Severity

We used ROC curves and graphical presentations to determine the optimal cut-off value for each peptide (Figure S1). We then analyzed the factors associated with the severity of SARS-CoV-2 infection. The results of this analysis are summarized in Table 3.

After adjustment, the analysis showed that a severe clinical presentation was more than three times more likely in patients over the age of 50 years (OR 3.5, 95% CI 1.05–13.36); 2.7 times more likely in male patients (OR 2.7, 95% CI 1.21–6.08) and 2.5 times more likely in patients with hypertension (OR 2.5, 95% CI 1.03–6.15). The severe clinical presentation was significantly associated with high levels of IgG reactivity to the SK27, NC3′ and M1 epitopes. Indeed, for OD values greater than 0.05 for SK27, the probability of having severe COVID-19 disease increased by 410%; the risk of severe infection was, thus, more than four times higher (OR 4.1, 95% CI 1.80–8.83). OD greater than 0.17 for NC3′ was associated with an almost four times higher risk of patients presenting severe clinical outcomes. Similarly, an OD greater than 0.9 for the M1 epitope was associated with higher severity, with a risk of developing the severe form almost seven times higher than that for an OD value below this threshold (OR 6.9, 95% CI 1.94–28.37). The probability of a severe COVID-19 outcome was four to seven times higher for values above the OD thresholds for SK27, NC3′ and M1 peptides than for OD values below these thresholds. However, for NS2, an OD > 2 was associated with a 90% lower risk of the severe clinical presentation than OD values below this threshold.

## 4. Discussion

Many studies have been performed to identify B-cell linear epitopes in the SARS-CoV-2 proteins [18,19,26,27,28,29,30,31,32,33,34,35,36,37]. Our study confirms previous findings, identifying the same overall regions. Surprisingly, only two epitopes were found on the S protein, located within the S1/S2 subdomain. No IgG response to peptide covering the receptor binding domain (RBD) was detected, despite this region being known to be highly immunogenic [38]. However, previous studies have reported similar results, suggesting that the RBD is rich in conformational epitopes but lacks linear epitopes [20]. In addition, we cannot exclude non-detection of peptide reactivity in our ELISA approaches, due to the short length of the peptides or their low efficiency to be adsorbed on the plates.

This study focused particularly on the possible association between disease severity and IgG response to these linear epitopes that could be analyzed by ELISA. The clinical factors associated with disease severity and mortality in COVID-19 patients have been studied in detail since the start of the pandemic. The clinical data for our patients are consistent with those reported in other studies, demonstrating that increasing age, being male, dyspnea, diabetes, hypertension, and obesity are associated with disease severity [39,40,41]. The magnitude of the humoral response is highly variable and has been shown to be positively correlated with disease severity [42]. Only two studies have described the epitopes eliciting this strong IgG response linked to severe clinical outcomes [19,28]. Both these studies identified immunodominant epitopes within the S and N proteins. We identified the same epitopes in our analysis, together with an additional epitope on the M protein, for which a strong humoral response was strongly correlated with disease severity. Indeed, we found that the IgG response elicited by the M1 epitope was responsible for the largest difference between disease severity groups. Patients with mild-to-moderate COVID-19 had weak antibody responses to M1, whereas patients with severe clinical presentations had very strong IgG responses to M1 (OR 6.9, 95% CI 1.94–28.37). Finally, we also analyzed the predicted B-cell linear epitopes of ORF8. We identified one epitope that elicited a strong humoral response in patients infected with SARS-CoV-2. ORF8 was recently identified as a novel secreted protein absent from all human pathogenic coronaviruses except SARS-CoV-2 [43]. It has been suggested that ORF8 plays a biological role in the pathogenesis of SARS-CoV-2, by mediating immune evasion via the downregulation of MHC-I molecules [44]. We therefore investigated whether patients with different severities of COVID-19 had different IgG responses to an epitope (NS2) identified in ORF8. We found large differences in reactivity between groups for this particular epitope. Logistic modeling revealed that the risk of developing severe COVID-19 decreased by 90% above the NS2 cut-off. However, the established cut-off is very high (OD > 2), suggesting that the IgG response was strong regardless of clinical presentation. This observation confirms that ORF8 is a unique and specific protein secreted by SARS-CoV-2. Our results suggest that patients with severe clinical outcomes have a weaker IgG response to ORF8 than patients with mild-to-moderate COVID-19 disease. We therefore provide new data for the NS2 epitope of the ORF8 protein, and describe a new epitope, M1, linked to COVID-19 severity. One limitation of this study is that we considered only short peptides. Longer peptides, which may retain some conformational information, may be more informative. However, the use of longer peptides would decrease the precision of identification for the epitopes involved. Another limitation of this study is that we could not have repetitions for all patients because of the restricted amount of plasma. However, when possible, plasma were tested in duplicate and OD obtained were very similar. In addition, a much larger cohort of COVID-19 patients with mild, moderate and severe disease will be required to strengthen these data.

The identification of serological markers of COVID-19 severity is an important goal, as it would facilitate profiling, making it possible to determine how viral responses differ between patients with different outcomes. The IgG response has been shown to be correlated with severity. This positive correlation raised the possibility that antibody-dependent enhancement (ADE) could, in some instances, contribute to the excessive immune response that exacerbates SARS-CoV-2 pathogenesis [45,46]. In this context, further studies on the immune response to the M1 and NS2 epitopes identified here will be useful, to determine the potential role of these epitopes in the immunopathogenicity of SARS-CoV-2 infection. Should these epitopes prove useful as clinical biomarkers of disease severity, ELISA-based assays would clearly be a valuable tool for monitoring them.

## Figures and Tables

**Table 1:** Characteristics of the patients included.

Clinical Data	Disease Severity			
	Mild N (%) 84 (39)	Moderate N (%) 42 (20)	Severe N (%) 87 (41)	Total N (%) 213 (100)
Median age (interquartile range) (years)	65 (45.7–87.0)	83.5 (58.5–89.5)	69 (61.0–75.0)	69.0 (55.0–84.0)
Male	27 (32)	15 (36)	57 (66)	99 (46)
Female	57 (68)	27 (64)	30 (34)	114 (54)
Cardiovascular disease	21 (25)	18 (43)	37 (42)	76 (35)
Hypertension	29 (34)	25 (60)	55 (63)	109 (51)
Diabetes mellitus	17 (20)	9 (21)	18 (21)	44 (21)
Lung disease	10 (12)	6 (14)	17 (19)	33 (16)
Renal insufficiency	5 (6)	7 (17)	9 (10)	21 (10)
Dialysis	0 (0.0)	0 (0)	1 (1)	1 (0.5)
Kidney transplantation	0 (0.0)	1 (2)	4 (5)	5 (2)
Liver failure	4 (5)	0 (0)	1 (1)	5 (2)
Obesity	12 (14)	10 (24)	30 (34)	52 (24)

Data are absolute numbers (%) or medians (interquartile range).

**Table 2:** IgG response to selected peptides.

Peptides of Interest	Disease Severity		
	Mild	Moderate	Severe
SK27	0.015 (0.000–0.065)	0.001 (0.000–0.1375)	0.342 (0.073–0.851)
SK28	0.138 (0.043–0.293)	0.124 (0.023–0.716)	0.591 (0.161–1.48)
NC2	0.237 (0.065–0.458)	0.273 (0.123–0.513)	0.432 (0.235–0.865)
NC3′	0.068 (0.005–0.649)	0.236 (0.037–2.101)	0.749 (0.221–2.522)
NC5′	0.532 (0.169–1.097)	0.662 (0.332–1.440)	1.052 (0.502–2.298)
M1	0.000 (0.000–0.235)	0.000 (0.000–0.085)	0.130 (0.000–1.012)
NS2	0.512 (0.072–1.216)	0.801 (0.476–1.319)	0.811 (0.467–1.138)

Data are expressed as the median (interquartile range).

**Table 3:** Clinical factors and B-cell epitopes associated with a severe clinical presentation, in multivariate logistic regressions analysis.

Variables of Interest	Univariate Analysis		Multivariate Analysis		
	Crude OR	*p*-Value	OR	95%CI	*p*-Value
***Clinical features***					
Age > 50 years	6.1	<10^−3^	3.5	1.05–13.36	0.049
Male	3.8	<10^−3^	2.7	1.21–6.08	0.02
Cardiovascular diseases	1.6	0.09	-	-	-
Hypertension	2.3	0.003	2.5	1.03–6.15	0.04
Chronic lung diseases	1.6	0.18	-	-	-
Diabetes mellitus	1.0	0.98	-	-	-
Obesity	2.6	0.01	-	-	-
Kidney chronic diseases	1.1	0.85	-	-	-
Liver failure	0.4	0.35	-	-	-
***Peptides***					
SK27 > 0.05	7.4	<10^−3^	4.1	1.80–9.83	<10^−3^
SK28 > 0.3	2.6	<10^−3^	-	-	-
NC2 > 0.1	4.2	0.001	-	-	-
NC3′ > 0.17	4.9	<10^−3^	3.9	1.70–9.22	0.002
NC5′ > 0.35	3.4	0.001	-	-	-
M1 > 0.9	9.1	<10^−3^	6.9	1.94–28.37	<10^−3^
NS2 > 2	0.1	0.06	0.1	0.002–0.63	0.03

## Data Availability

The data presented in this study are available on request from the corresponding author. The data are not publicly available due to privacy issues.

## Supplementary Materials

The following supporting information can be downloaded at: https://www.mdpi.com/article/10.3390/jcm11020405/s1, Figure S1: Graphic determination of optimal OD cut-off for each peptide, Table S1: Peptide sequences of putative immunodominant SARS-CoV-2 IgG linear B-cell epitopes, Table S2: Peptides of interest: selection.
