# Supplementary material for: ELISA-Based Analysis Reveals an Anti-SARS-CoV-2 Protein Immune Response Profile Associated with Disease Severity
Source: J Clin Med. 2022 Jan 14;11(2):405. doi: 10.3390/jcm11020405 (PMC8781066; doi:10.3390/jcm11020405)
Supplement: Supplementary file 1 [file jcm-11-00405-s001.zip › jcm-1532072-supplementary.pdf]

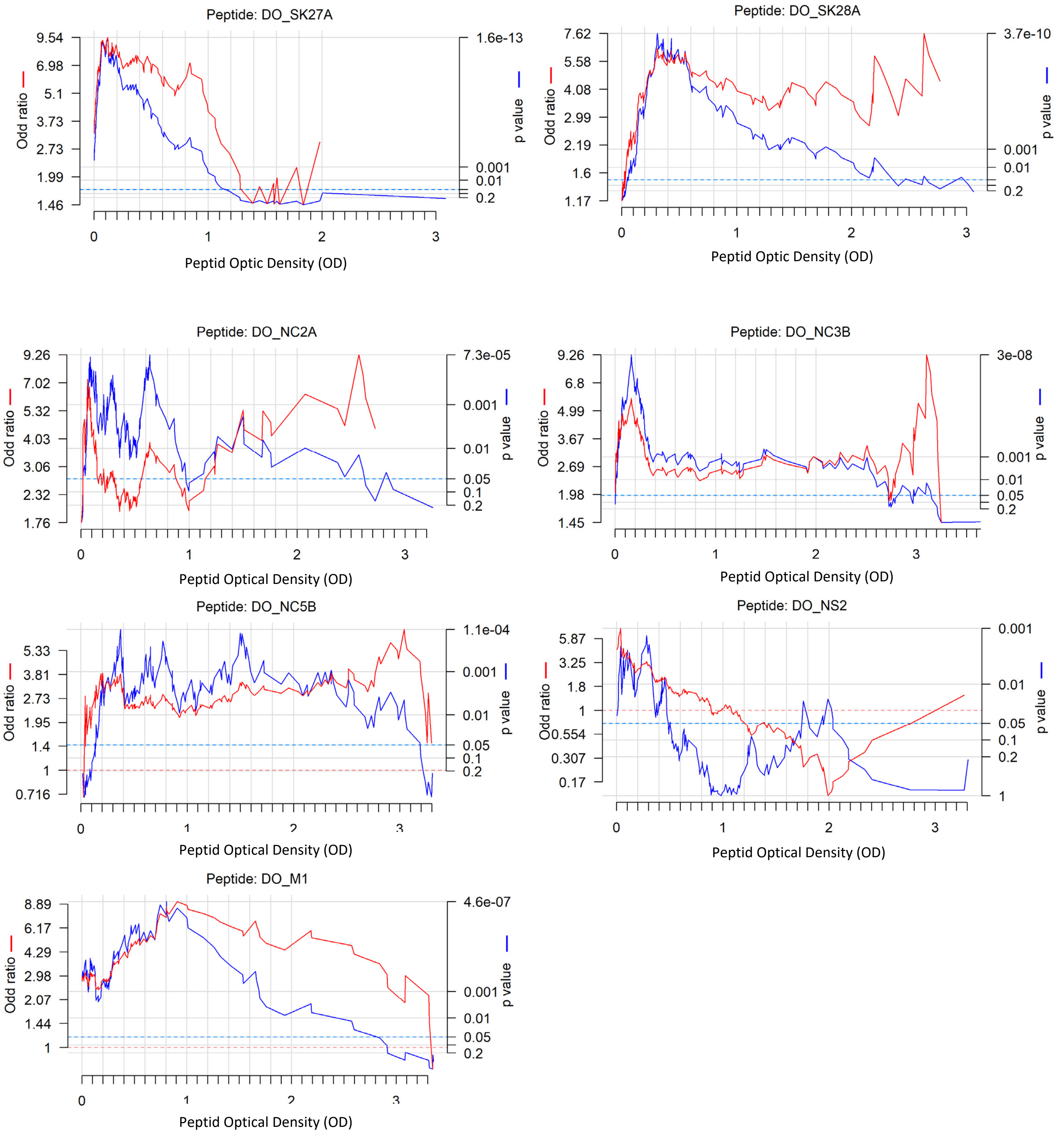

Figure S1. Graphic determination of optimal cut-off of OD for each peptide. Each cut-off has been chosen accordingly to the best balance between odds ratio of severity and its p-value. We proposed a graphical plot that illustrates the DO ability to classify the COVID-19 disease as severe according to the variations in the DO value distribution, giving a discrimination threshold = cut-off (on the same basis as ROC curves).

Table S1. Peptide sequences of putative immunodominant SARS-CoV-2 IgG linear B-cell epitopes.

Peptide ID	Position	Amino acid sequence
SK1	27-46	AYTNSFTRGVYYPDKVFRSS
SK2	59-78	FSNVTWFHAIHVSGTNGTKRF
SK3	75-94	GTKRFDNPVLPFNDGVYFAST
SK4	91-110	YFASTEKSNIIRGWIFGTTLD
SK5	107-126	GTTLDSKTQSLLIVNNATNVV
SK6	123-142	ATNVVIKVCEFQFCNDPFLGV
SK7	139-158	PFLGVYYHKNNKSWMESEFRV
SK8	155-174	SEFRVYSSANNCTFEYVSQPF
SK8'	150-180	KSWMESEFRVYSSANNCTFEYVSQPFLMDLE
SK9	171-190	VSQPFLMDLEGKQGNFKNLRE
SK10	187-206	KNLREFVFKNIDGYFKIYSKH
SK11	203-222	IYSKHTPINLVRDLPQGFSAL
SK12	236-255	TRFQTLLALHRSYLTPGDSSS
SK13	252-271	GDSSSGWTAGAAAYYVGYLQ
SK13'	247-276	SYLTPGDSSSGWTAGAAAYYVGYLQPRTFL
SK14	328-347	RFPNITNLCPFGEVFNATRFA
SK15	344-363	ATRFASVYAWNRKRISNCVAD
SK16	360-379	NCVADYSVLYNSASFSTFKC
SK16'	355-385	RKRISNCVADYSVLYNSASFSTFKCYGVSPT
SK17	376-395	TFKCYGVSPTKLNDLCFTNVY
SK18	392-411	FTNVYADSFVIRGDEVRQIAP
SK19	408-427	RQIAPGQTGKIADYNYKLPDD
SK18-19	391-428	CFTNVYADSFVIRGDEVRQIAPGQTGKIADYNYKLPDD
SK20	424-443	KLPDDFTGCVIAWNSNNLDSK
SK22	456-475	FRKSNLKPFERDISTEIYQAG
SK23	472-491	IYQAGSTPCNGVEGFNCYFPL
SK23'	467-497	DISTEIYQAGSTPCNGVEGFNCYFPLQSYGF
SK24	488-507	YFPLQSYGFQPTNGVGYQPYR
SK25	504-523	GYQPYRVVVLSFELLHAPATV
SK25'	499-529	PTNGVGYQPYRVVVLSFELLHAPATVCGPKK
SK26	520-539	APATVCGPKKSTNLVKNKCVN
SK27	549-568	TGVLTESNKKFLPFQQFGRD
SK28	571-590	DTTDAVRDPQTLEILDITPCSF
SK28'	566-597	GRDIADTTDAVRDPQTLEILDITPCSFGGVSV
SK29	700-717	GAENSVAYSNNSIAIPTNFT
SK30	782-801	FAQVKQIYKTPPIKDFGGFNF
SK31	798-817	GGFNFSQILPDPSKPSKRSF
SK32	877-896	LLAGTITSGWTFGAGAALQIP
SK33	893-912	ALQIPFAMQMAYRFNGIGVTQ
SK34	909-928	IGVTQNVLYENQKLIANQFN
SK34'	904-940	YRFNGIGVTQNVLYENQKLIANQFNSAIGKIQDSLSS
SK35	1067-1086	YVPAQEKNFTTAPAICHDGKA
SK35'	1062-1092	FLHVTYVPAQEKNFTTAPAICHDGKAHFPRE
SK36	1083-1102	HDGKAHFPREGVFVSNGTHWF
SK37	1099-1118	GTHWFVTQRNFYEPQIITTDN
SK39	1131-1150	GIVNNTVYDPLQPELDSFKE
NC1	22-42	DSTGSNQNGERSGARSKQRRP
NC2	87-108	YRRATRRIRGGDGKMKDLSPRW
NC2'	82-112	DQIGYYRRATRRIRGGDGKMKDLSPRWYFYY
NC3	153-172	NNAAIVLQLPQGTTLPKGFY
NC3'	148-177	TRNPANNAAIVLQLPQGTTLPKGFYAEGSR
NC4	177-198	RGGSQASSRSSSRSRNSSRNST
NC5	371-390	DKKKKADETQALPQRQKKQQ
NC5'	366-395	TEPKKDKKKKADETQALPQRQKKQQTVTLL
NC6	1-23	MSDNGPQNQRNAPRITFGGPSDS
NC8	242-274	QGQTVTKKSAAEASKKPRQKRTATKAYNVTQAF
M1	1-19	MADSNGTITVEELKKLLEQ
NS1	24-44	SCTQHQPYVVDDPCPIHFYSK
NS2	38-59	PIHFYSKWYIRVGARKSAPLIE
NS3	61-82	CVDEAGSKSPIQYIDIGNYTVS
SKS-36	-	CESFATGVHFHHGDWRNPVGK

Table S2. Peptides of interest: selection**.** Peptides giving a mean optical density (OD) superior to 0.3 and at least 5 fold superior to the OD obtained with the negative control were selected for further analysis.

**Peptide ID**	**OD (Mean ± SD)**	
	**COVID – pool**	**COVID +**
SK1	0.047 ± 0.067	0.056 ± 0.114
SK2	0.072 ± 0.050	0.073 ± 0.115
SK3	0.033 ± 0.057	0.055 ± 0.090
SK4	0.099 ± 0.101	0.086 ± 0.112
SK5	0.007 ± 0.010	0.059 ± 0.099
SK6	0.020 ± 0.030	0.073 ± 0.109
SK7	0.072 ± 0.119	0.075 ± 0.084
SK8	0.008 ± 0.020	0.102 ± 0.181
SK8’	0.005 ± 0.010	0.050 ± 0.085
SK9	0.056 ± 0.046	0.101 ± 0.151
SK10	0.087 ± 0.128	0.126 ± 0.140
SK11	0.031 ± 0.059	0.086 ± 0.184
SK12	0.066 ± 0.063	0.082 ± 0.084
SK13	0.023 ± 0.027	0.099 ± 0.124
SK13’	0.002 ± 0.008	0.018 ± 0.061
SK14	0.122 ± 0.111	0.112 ± 0.125
SK15	0.170 ± 0.070	0.320 ± 0.238
SK16	0.012 ± 0.029	0.101 ± 0.166
SK16’	0.019 ± 0.051	0.020 ± 0.047
SK17	0.034 ± 0.05	0.152 ± 0.186
SK18	0.084 ± 0 .094	0.075 ± 0.097
SK19	0.022 ± 0.021	0.117 ± 0.170
SK18-19	0.016 ± 0.014	0.042 ± 0.095
SK20	0 .062 ± 0.088	0.078 ± 0.129
SK22	0.047 ± 0.060	0.129 ± 0.263
SK23	0.009 ± 0 .014	0.128 ± 0.223
SK23’	0.00043 ± 0.002	0.062 ± 0.161
SK24	0.077 ± 0.101	0.123 ± 0.232
SK25	0.027 ± 0.057	0.018 ± 0.038
SK25’	0.032 ± 0.016	0.074 ± 0.108
SK26	0.205 ± 0.065	0.464 ± 0.409
SK27	0.024 ± 0.027	0.335 ± 0.450
SK28	0.175 ± 0.063	0.748 ± 0.746
SK28’	0.034 ± 0.076	0.349 ± 0.425
SK29	0.084 ± 0.103	0.085 ± 0.110
SK30	0.063 ± 0.051	0.328 ± 0.556
SK31	0.134 ± 0.223	0.112 ± 0.158
SK32	0.136 ± 0.178	0.086 ± 0.134
SK33	0.017 ± 0.042	0.058 ± 0.144
SK34	0.056 ± 0.095	0.036 ± 0.084
SK34’	0.00018 ± 0.001	0.055 ± 0.224
SK35	0.164 ± 0.167	0.123 ± 0.198
SK35’	0.014 ± 0.032	0.061 ± 0.111
SK36	0.222 ± 0.293	0.079 ± 0.118
SK37	0.041 ± 0.045	0.074 ± 0.107
SK39	0.087 ± 0.121	0.093 ± 0.159
NC1	0.003 ± 0.006	0.048 ± 0.125
NC2	0.101 ± 0.132	0.605 ± 0.664
NC2’	0.009 ± 0.034	0.212 ± 0.304
NC3	0.002 ± 0.007	0.454 ± 0.700
NC3’	0.017 ± 0.031	1.063 ± 1.187
NC4	0.021 ± 0.016	0.122 ± 0.257
NC5	6.10^{-5}± $2.46.10^{-4}$	0.253 ± 0.438
NC5’	0.082 ± 0.038	1.103 ± 0.856
NC6	0.005 ± 0.019	0.088 ± 0.284
NC8	0.202 ± 0.058	0.237 ± 0.286
M1	0.032 ± 0.105	0.489 ± 0.856
NS1	0.057 ± 0.202	0.068 ± 0.174
NS2	0.176 ± 0.084	1.275 ± 0.725
NS3	0.037 ± 0.078	0.208 ± 0.554
